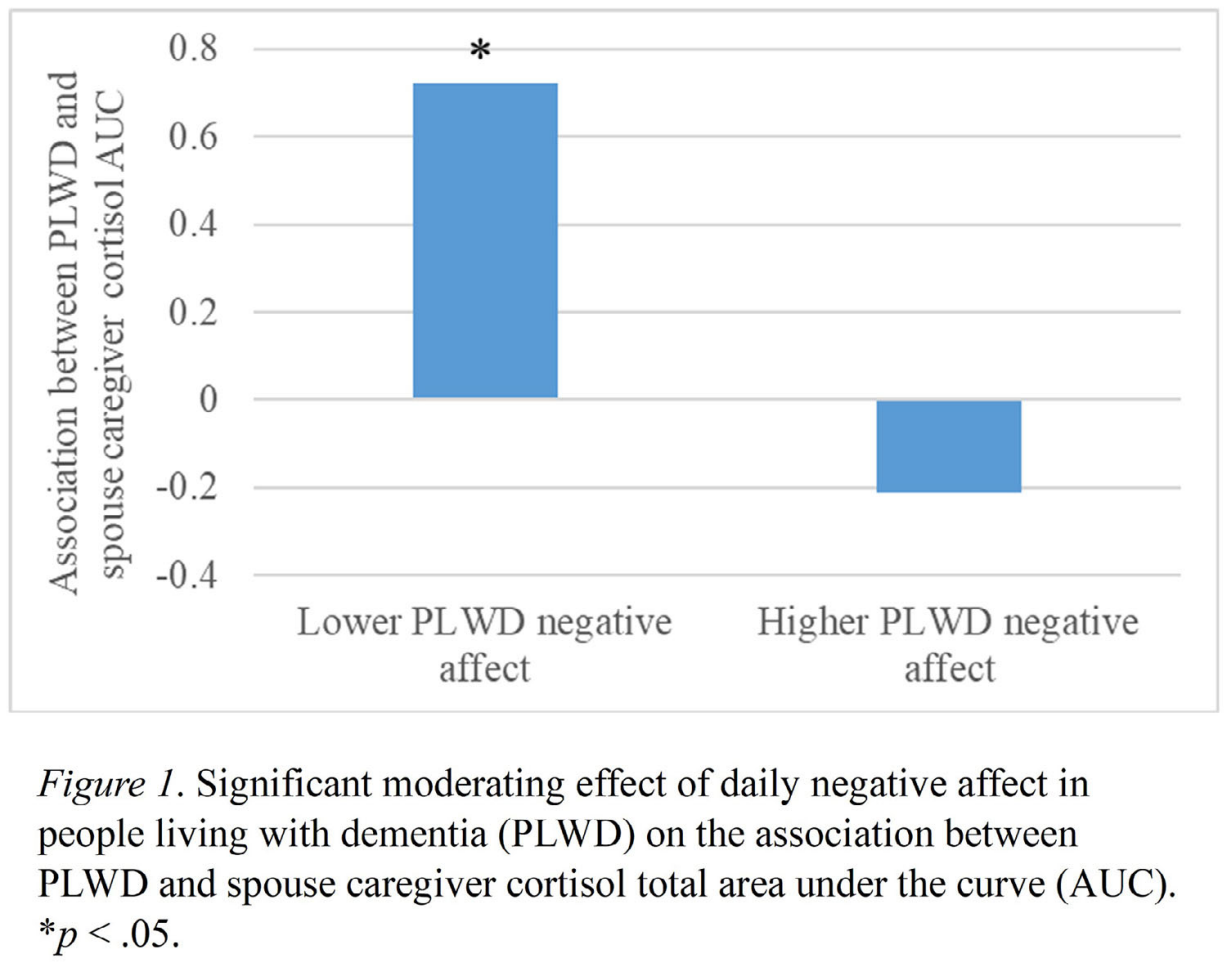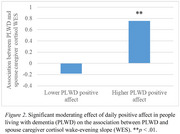# Daily Affect Within Care Dyads: Links to Cortisol and Alpha‐Amylase Synchrony in Couples Living With Early‐Stage Dementia

**DOI:** 10.1002/alz70858_104185

**Published:** 2025-12-26

**Authors:** Courtney A. Polenick, Angela Turkelson, Charity Garner, Yin Liu, Kira S. Birditt

**Affiliations:** ^1^ University of Michigan, Ann Arbor, MI, USA; ^2^ Utah State University, Logan, UT, USA

## Abstract

**Background:**

Couples often show synchrony, or linked patterns, in their reactivity to daily stress. However, we know little about factors that predict synchrony in stress reactivity among couples living with Alzheimer's disease and related dementias (ADRD). We evaluated how daily affect reported by people living with dementia (PLWD) and their spouse caregivers predicts synchrony in diurnal cortisol and alpha‐amylase rhythms among heterosexual couples living with early‐stage ADRD.

**Method:**

Participants included 34 PLWD (*M* = 71.2 years, *SD* = 7.5, range = 52‐85 years) and their spouse caregivers (*M* = 69.5 years, *SD* = 8.7, range = 49‐88 years) who completed 7 days of brief morning and evening phone interviews and collected saliva samples (four times per day) during four of those days. Outcomes included the awakening response (AR; awakening to 30 min post‐awakening), wake‐evening slope (WES; 30‐min post‐awakening to bedtime), and total area under the curve (AUC) for cortisol and alpha‐amylase. Multilevel models controlled for weekend day and age.

**Result:**

On days when PLWD reported lower negative affect, PLWD and caregivers showed a positive association in their cortisol AUC (*b* = 0.72, *SE* = 0.30, *p* = .016) and cortisol WES (*b* = 0.81, *SE* = 0.27, *p* = .003). On days when PLWD reported higher positive affect, PLWD and caregivers showed a positive association in their cortisol WES (*b* = 0.76, *SE* = 0.25, *p* = .003). On days when caregivers reported higher negative affect, PLWD and caregivers showed a positive association in their alpha‐amylase AUC (*b* = 0.52, *SE* = 0.17, *p* = .002). Finally, on days when caregivers reported lower positive affect, PLWD and caregivers showed a positive association in their alpha‐amylase AUC (*b* = 0.68, *SE* = 0.18, *p* < .001). Daily affect did not predict synchrony in cortisol or alpha‐amylase AR or alpha‐amylase WES.

**Conclusion:**

Overall, when PLWD reported better daily affect, PLWD and spouse caregivers were synchronized in cortisol AUC and WES. By contrast, when caregivers reported worse daily affect, PLWD and caregivers were synchronized in alpha amylase AUC. These findings inform targets of interventions to promote the well‐being of both care dyad members.